# Obesity and Hypogonadism—A Narrative Review Highlighting the Need for High-Quality Data in Adolescents

**DOI:** 10.3390/children6050063

**Published:** 2019-05-01

**Authors:** Tasnim Mushannen, Priscilla Cortez, Fatima Cody Stanford, Vibha Singhal

**Affiliations:** 1 Neuroendocrine Unit, Massachusetts General Hospital and Harvard Medical School, Boston, MA 02114, USA; tam2029@qatar-med.cornell.edu (T.M.); priscillacortez@email.arizona.edu (P.C.); 2 Weill Cornell Medicine–Qatar, Education City, Doha 24144, Qatar; 3 University of Arizona, Tucson, AZ 85721, USA; 4 Pediatric Endocrinology, Massachusetts General Hospital and Harvard Medical School, Boston, MA 02114, USA; 5 MGH Weight Center, Massachusetts General Hospital, Boston, MA 02114, USA

**Keywords:** obesity, adolescents, hypogonadism, testosterone

## Abstract

The prevalence of obesity continues to rise in adult and pediatric populations throughout the world. Obesity has a direct impact on all organ systems, including the reproductive system. This review summarizes current knowledge about the effects of obesity on the male reproductive system across age, highlighting the need for more data in children and adolescents. Male hypogonadism is commonly seen in patients with obesity and affects the onset, duration, and progression of puberty. Different pathophysiologic mechanisms include increased peripheral conversion of testosterone to estrone and increased inflammation due to increased fat, both of which lead to suppression of the hypothalamic-pituitary-gonadotropin (HPG) axis and delayed development of secondary sexual characteristics in adolescent males. Evaluation of the HPG axis in obesity includes a thorough history to exclude other causes of hypogonadism and syndromic associations. Evaluation should also include investigating the complications of low testosterone, including increased visceral fat, decreased bone density, cardiovascular disease risk, and impaired mood and cognition, among others. The mainstay of treatment is weight reduction, but medications such as testosterone and clomiphene citrate used in adults, remain scarcely used in adolescents. Male hypogonadism associated with obesity is common and providers who care for adolescents and young adults with obesity should be aware of its impact and management.

## 1. Introduction

Obesity has become a prominent chronic disease worldwide. In less than 45 years, the prevalence of obesity has tripled [1]. Currently, more than 1.9 billion adults and 381 million children and adolescents have overweight or obesity [1,2]. The prevalence of obesity in the United States is almost 40% in adults and 19% in children and adolescents [3]. This 19% translates into 13.7 million children and adolescents with obesity [3].

Adolescent obesity is defined as a body mass index (BMI) equal to or greater than the age and gender specific 95th percentile [4]. Factors leading to the development of adolescent obesity include genetic, neuroendocrine, socio-economic, psychological, metabolic and environmental factors [4]. Obesity negatively impacts every organ system in the body and is a significant risk factor for co-morbid disease, cardiovascular disease, type 2 diabetes mellitus (T2DM), and cancer [4]. Obesity also affects the reproductive system and is associated with polycystic ovarian syndrome (PCOS) in women and testosterone deficiency, causing hypogonadism in men. 

## 2. Methods

This is a narrative review of the current literature on hypogonadism in males with obesity. The literature included in this study was identified by search on the following databases: PubMed, Scopus, Google Scholar, and ProQuest. The keywords used include: “obesity”, “adolescent”, “hypogonadism”, “male”, “testosterone”, “inflammation”, “insulin resistance”, “puberty”, “adult height”, “bone age”, “fatigue”, “depression”, “bone density”, “clomiphene citrate”, “hCG”. Keywords were used either alone or in combination.

## 3. Definition and Prevalence of Male Hypogonadism

The prevalence of hypogonadism is significantly greater in males with obesity than in normal-weight controls. This has been well characterized in adults with obesity, but data are less robust in children and adolescents with obesity. Normal total testosterone (TT) levels in adult males range from 270 to 1070 ng/dL and it varies by age and pubertal stage in developing males [5,6]. There is no universal definition of hypogonadism, even in adults, with some studies using TT and others using free testosterone (FT) or both [7]. A 2013 study found the prevalence of hypogonadism in 161 adult males with a median age of 45 years to be around 32% and also showed that 75% of subjects with severe obesity (BMI > 40 kg/m^2^) had hypogonadism [8]. The largest study evaluating hypogonadism in 2162 males 45 years and older reported a prevalence of 38.7% based on the definition of TT < 300 ng/dL [9]. A more recent study that used either TT or free testosterone (FT) to define hypogonadism (using a normal range of 288–894 ng/dL for TT and 6.5–18.3 ng/dL for FT) showed a prevalence of 45% based on either low TT or FT values. When considering only low, T.T.; the prevalence was 44%, and when considering only low, F.T.; it was 34% [10]. Furthermore, the cut-offs for hypogonadism are likely impacted by the specific assay used to measure total or free testosterone and the timing of the day. The multicentric European Male Aging Study (EMAS), correlated hypogonadism symptoms with decreased testosterone levels in 3219 men older than 40 years and created a standardized criteria for diagnosing late-onset hypogonadism [11]. They came up with the criteria of having at least three sexual symptoms along with both TT < 11 nmol/L (317 ng/dL) and FT < 220 pmol/L (6.34 ng/dL) [11]. Using this definition, the prevalence of late-onset hypogonadism in their study sample was 2.1%, while the prevalence of having a TT level < 11 nmol/L was 17.0% [11].

Very few studies report the prevalence of hypogonadism in adolescent males with obesity. One study demonstrated a 33% prevalence among 14–35 year old males with obesity based on abnormally low FT concentrations [12]. Another study reported a 40% prevalence among adolescent males with obesity based on a FT concentration <6.63 ng/dL [13]. In a study of male adolescents and young adults (18–35 years old) with T2DM, 33% were considered to have hypogonadism if they had an FT lower than the standard normal range of 8.01 to 22.39 ng/dL, while 58% were considered to have hypogonadism when using the respective age-specific FT normal range [14]. Thus, the prevalence of hypogonadism in adolescent and young adult males with obesity varies anywhere from 30–60% based on different criteria used for the diagnosis and severity of obesity.

## 4. Pathogenesis of Hypogonadism

### 4.1. Hypothalamic-Pituitary-Gonadotropin (HPG) Axis

Under normal conditions, following the onset of puberty, the hypothalamus releases gonadotropin-releasing hormone (GnRH) to stimulate the pituitary gland to produce and release luteinizing hormone (LH) and follicle-stimulating hormone (FSH) [15]. LH stimulates Leydig cells, which surround the seminiferous tubules to produce testosterone [15]. FSH stimulates Sertoli cells of the testis to provide support and nutrition to the developing sperm in the testis [15].

**Figure 1:**
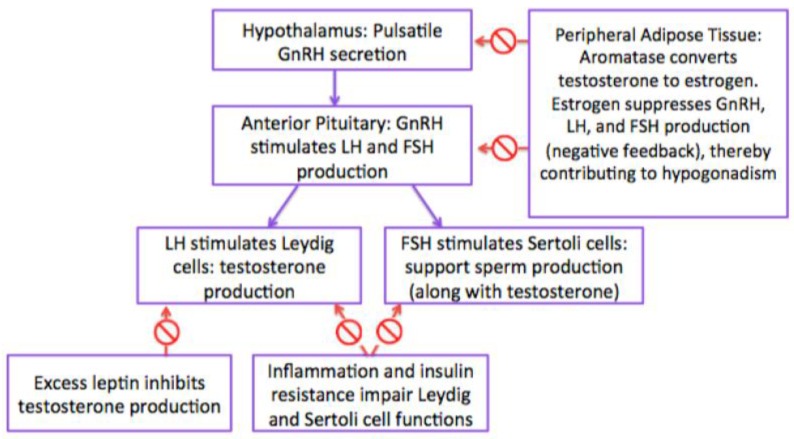
Hypothalamic-Pituitary-Gonadotropin (HPG) Axis.

Aromatase is a cytochrome P450 enzyme present in adipose tissue that converts androgens, including testosterone, into estrogens. In obesity, because of the excess adipose tissue, there is increased conversion of testosterone to estradiol and androstenedione to estrone [16]. Through a negative feedback mechanism, estrogens inhibit GnRH release from the hypothalamus, as well as LH and FSH release from the pituitary, resulting in overall decreased testosterone production (Figure 1) [16]. This is important during puberty, when testosterone is needed for development of the testicles, production of sperm, and development of secondary sexual characteristics [16].

### 4.2. Estrogen

While most studies show that obesity is associated with elevated estradiol levels in males, some studies challenge these findings [12,13,17,18,19] One study demonstrated significantly higher estradiol levels in male adolescents with obesity vs. normal-weight adolescents during both pre-pubertal and pubertal periods [18]. Within pubertal males with obesity, BMI was a positive predictor of estradiol levels, suggesting that estradiol levels increase with increasing severity of obesity [18]. However, other studies report no significant difference in estradiol levels between boys with and without obesity [13,17,19]. Some studies even show decreased estrogen [12,13]. The inconsistency in data may arise from different populations being studied based on age, puberty, obesity severity and different estradiol assays. 

### 4.3. Gonadotropins (Follicle Stimulating Hormone and Luteinizing Hormone) and Testosterone

FSH and LH are hormones that regulate sexual development in both males and females. In males, FSH stimulates Sertoli cells to support sperm production and produce androgen-binding protein, which binds testosterone and increases its levels in the seminiferous tubule, which further promotes sperm production [20]. LH is necessary for the production of testosterone by Leydig cells [20]. Thus, decreased FSH and LH result in decreased stimulation of Sertoli and Leydig cells, resulting in inadequate support and nourishment to developing spermatozoa and decreased testosterone production, respectively [20]. This effect is seen in males with obesity due to the suppression of the HPG axis seen in obesity. In fact, a recent study suggested a causal relationship between BMI and serum testosterone levels. The researchers found in 7000 males with obesity that the genetic risk score for BMI was inversely related to testosterone levels, even after controlling for age and smoking status [21]. The genetic predisposition to having elevated BMI resulted in lower testosterone level, suggesting a causal relationship of obesity in hypogonadism. Reductions in testosterone that result from obesity-related hypogonadism contribute to poor spermatogenesis and impaired development and progression of sexual characteristics [20].

### 4.4. Sex Hormone Binding Globulin (SHBG)

A crucial factor that impacts hormone levels and regulates how estrogen and androgens interact is the level of sex hormone-binding globulin (SHGB). SHGB is a carbohydrate-rich beta-globulin protein produced by hepatocytes, and it binds to testosterone with high affinity and prolongs its metabolic clearance [22]. There is a positive association between the level of SHGB and the level of total testosterone in the body. SHGB levels in boys with obesity are lower at all stages of development from pre-puberty to post-puberty compared to normal-weight boys who experience higher SHGB levels [23]. These lower levels contribute to the reduction in total testosterone seen in pubertal boys with obesity [23,24].

Free testosterone is the bioavailable form of testosterone which is not bound to SHBG, and it is a better indicator of androgen activity than total testosterone [24]. The data on the levels of free testosterone in males with obesity—in both adolescents and adults—is variable with most studies showing adequate levels [22,23,24].

### 4.5. INSL3

INSL3 is a peptide hormone secreted from mature Leydig cells and reflects their functionality [25]. INSL3 plays a role in the development of male reproductive organs during embryogenesis, as well as in the survival of male germ-cells later in life [17]. INSL3 mRNA levels increase during spermatogenesis and germ-cell maturation, further illustrating its role in gonadal function [26]. Studies show that INSL3 levels are even more sensitive for Leydig cell impairment than androgen levels, especially in cases of male infertility in which testosterone levels may be preserved while INSL3 levels are reduced [17,26]. When comparing INSL3 levels in adolescent males with and without obesity, significantly lower levels were found in boys with obesity, suggesting significantly reduced Leydig cell function in these boys [17]. This result was found for boys with obesity in both early (Tanner 2) and late (Tanner 4) stages of puberty [17].

### 4.6. Adipokines (Leptin)

Leptin is a hunger-suppressing hormone produced by adipose tissue [27]. It is one of many hormones involved in appetite, body weight regulation, and energy homeostasis [27]. These systems are dysregulated in obesity [27]. Specifically, low leptin levels increase GnRH release and affect the pulsatility, but high leptin levels have the opposite effect and inhibit its release [8]. Nonetheless, both low and high leptin levels decrease GnRH function, impacting the HPG axis in response [28]. There must be an optimal level of leptin signaling to the brain to allow GnRH pulses to stimulate the gonadotropes for optimal FSH and LH secretion [28]. Obesity is associated with increased leptin levels, which suggests another pathophysiologic mechanism to hypogonadism in adolescents with obesity [16,27]. Furthermore, it has been shown that Leydig cells express leptin receptors, and the expression level of the receptors is inversely related to testosterone level [17,29,30]. The effect of excess leptin on Leydig cells is suppression of testosterone production; thus, elevated leptin in adolescents with obesity contributes to hypogonadism [17,29,30]. Moreover, one study in adolescent males with obesity showed a negative correlation between INSL3 and leptin levels (*r* = −0.468, *p* = 0.001), further illustrating leptin’s association with reduced Leydig function and hypogonadism [17,31]. 

### 4.7. Insulin Resistance

Insulin resistance is also associated with hypogonadism. In fact, the association between obesity and hypogonadism in adult males is even stronger in those with T2DM than in those without T2DM [32]. The association between insulin resistance and hypogonadism in adolescent males is not as well defined. Nevertheless, one study comparing adolescent males 12 to 19 years old with and without obesity and T2DM showed that insulin sensitivity was an independent predictor of testosterone levels [32]. Indeed, testosterone levels in adolescent males were not only directly related to BMI and weight, but also to the degree of insulin resistance [33]. This result was found regardless of Tanner stage [32]. Another study in 50 adolescent males found a similar result, with free testosterone concentrations negatively correlated with the degree of insulin resistance [34]. Insulin resistance and hyperinsulinemia are also associated with low SHGB, LH, FSH, and total testosterone levels in adult males [12].

Other studies demonstrate a direct association between insulin resistance and male obesity secondary hypogonadism (MOSH). In one study, the prevalence of MOSH was 25–40% in adult and adolescent males with T2DM [14,33]. Furthermore, MOSH is rare in type 1 diabetics, who do not typically exhibit insulin resistance, further illustrating the contribution of insulin resistance and not hyperglycemia to hypogonadism [14,33].

### 4.8. Inflammation

Contrary to past beliefs that adipose tissue is metabolically inactive, adipocytes secrete a myriad of adipose-specific and systemic inflammatory factors [35,36]. In a study on 671 children and adolescents with a mean age of 13.3 ± 2.7 years, higher levels of the pro-inflammatory cytokine interleukin-6 (IL-6) were directly related to adipose tissue insulin resistance [36]. Another study found a positive correlation between BMI and the number of neutrophils (one of the major inflammatory cells) in the blood [37]. This subclinical inflammatory state is associated with reduced sex hormone levels in both adults and adolescents [38]. Studies show that obesity increases the inflammatory marker tumor necrosis factor (TNF)-alpha as well as inflammatory cells in the testicles that contribute to Leydig cell damage, inhibition of LH signaling, and thus reduced testosterone production [16,39]. Pro-inflammatory cytokines disrupt seminiferous epithelium and epididymal tissue, reducing sperm quality, and quantity as well [16]. Spermatogenesis is also impaired due to the increased inflammation and heat produced by adipose tissue in areas surrounding the scrotum [16]. Furthermore, inflammatory cytokines reduce GnRH secretion from the hypothalamus, further contributing to reduced testosterone levels [40].

### 4.9. Variations in Genes

Although there is an association between obesity and male hypogonadism, not every male with obesity has hypogonadism, suggesting that there may be higher susceptibility or other contributing factors to the development of hypogonadism. Recent studies suggest that genetic variation may contribute to the development of hypogonadism in male patients with obesity [41]. In a study where genetic alleles that increase the risk of isolated hypogonadotropic hypogonadism (IHH) were studied in 160 male patients, the prevalence of the genetic variants that predispose to (IHH) was greater in IHH patients with obesity than in controls [41]. Thus, underlying genetic variations in males with obesity may play a role in developing hypogonadism.

Another study found that the prevalence of obesity in patients with adult-onset IHH was twice the prevalence of obesity in the general population [42]. The study suggested that obesity is an acquired factor that significantly contributes to HPG suppression in individuals who are already genetically susceptible to developing IHH [42].

### 4.10. Growth and Puberty in Males with Obesity

#### 4.10.1. Timing of Puberty

Data on the sexual maturation of male adolescents with obesity is conflicting. While most studies report delayed puberty in males with obesity, some report normal or advanced puberty [18]. A handful of studies report normal gonadal development despite low testosterone levels at puberty [18,43]. One study showed no significant difference in testicular volume of pubertal male adolescents with obesity and those without obesity based on Tanner stage [18]. On the other hand, a large Swedish longitudinal study of 4488 subjects showed earlier onset of puberty (defined as time to reach peak height velocity); another study in Denmark showed similar results [44,45]. An additional study reported early puberty based on age at voice break [46]. Both of these studies have evaluated the late stages of puberty and not the onset. Nevertheless, most studies show delayed puberty in male adolescents with obesity [47,48]. A recent 4-year longitudinal study showed that after separating 401 boys into 3 groups based on, B.M.I.; those in the highest BMI group had the greatest risk of delayed puberty (as determined by genitalia development) than those in the lowest BMI group [48]. In another cross-sectional study in 10–16 year-olds, there were significantly lower percentages of adolescent males with obesity who achieved pubarche and voice break compared to normal weight controls [47]. These data suggest that in males with obesity, while the pubertal onset as assessed by genitalia development may be delayed, the timing of pubertal completion is similar to their normal weight peers. Nonetheless, the genetic variability found in self-limited delayed puberty may contribute to the delay in pubertal onset associated with obesity as well. Zhu and colleagues found that self-limited delayed puberty has genetic associations with congenital HH particularly rare variants in the TAC3, TACR3, and IL17RD genes which may lead to a deficiency or insensitivity to GnRH (Table 1) [49]. Pubertal delay associated with obesity, may also be partially attributable to these genetic variations and need to be further explored.

#### 4.10.2. Bone Age Advancement and Final Adult Height

In a study with pre-pubertal children with or without obesity and/or premature adrenarche, obesity was highly correlated with bone age advancement [23]. On the other hand, weight loss in a group of pediatric patients with obesity was associated with a significant reduction in bone age advancement [23]. Obesity in males is associated with advanced bone maturation, but this is not synonymous with earlier pubertal development when based on genitalia development [18,24].

Heights of both pre-pubertal and pubertal male adolescents with obesity are higher than those of normal BMI controls in some studies [18,50]. However, studies also show that final adult height is reduced in adolescents with obesity compared to adolescents with normal weight [51,52]. Although height gain is greater in children (younger age) with obesity, adolescents with obesity have bone age advancement and hence stop growing sooner than adolescents without obesity [44,52,53,54]. Moreover, the height gained during puberty is also less in adolescents with higher BMIs [55]. A more recent study also found significant differences in the median adult heights of adolescent males with and without obesity (174 cm vs. 176 cm, respectively; *p* = 0.025) [52]. Thus, while taller at the onset of puberty, males with obesity have smaller height gains during puberty and may eventually have shorter adult heights than genetic potential due to early skeletal maturity.

## 5. Syndromes Associated with Obesity and Hypogonadism

The most common cause of male hypogonadism in adults is obesity [8]. Male obesity-associated secondary hypogonadism (MOSH) is a diagnosis of exclusion, which is made after the primary causes of hypogonadism have been ruled out. It is defined as a syndrome of the inability to produce adequate amounts of testosterone and/or sperm in males with obesity [20,56]. Table 1 details other syndromes associated with hypogonadism. Males with primary hypogonadism (most commonly due to disorders affecting the testes) will have low testosterone with high LH and, F.S.H.; while males with secondary hypogonadism (most commonly due to disorders affecting the hypothalamus or anterior pituitary) will have low testosterone levels with low or normal LH and FSH [15] (Table 1).

## 6. Clinical Presentation

Hypogonadism may present in puberty or adulthood [56]. During puberty, the main features of hypogonadism are those of delay in the onset or progression of secondary sexual trait development, including a delay in testicular maturation, penile development and reduced pubic hair growth [15,56]. Male adolescents with hypogonadism may present with body image issues from increased abdominal fat accumulation and the development of breast tissue (gynecomastia) which results from an imbalance in the ratio of estrogen to testosterone levels in males with obesity [15,56,57,58].

Adolescents who acquire hypogonadism in late stages of puberty may experience erectile dysfunction, blunted libido, and/or abnormally low semen volumes, as seen in adults [15,56]. Many cases of male hypogonadism result in infertility [56]. There are no longitudinal studies to date to evaluate the impact of childhood obesity with or without its resolution in adulthood on male infertility.

## 7. Evaluation

### 7.1. Diagnosis

Adolescents with hypogonadism present with few symptoms, unlike adults who may manifest many of the mentioned symptoms, and thus high clinical suspicion and further laboratory testing are required for male adolescent hypogonadism. A fasting early morning total testosterone is the initial test performed as adolescent males with obesity have significantly lower TT than adolescents without obesity [13,15]. TT includes T bound to SHGB (40–70%), albumin (20–50%), and free. In males who are 19 years and older, two successive measurements of TT concentrations <250–300 ng/dL along with clinical signs of hypogonadism are required for diagnosis [15]. In adolescents, age-specific testosterone ranges are more commonly used [59]. The levels of other hormones, including SHGB (done to estimate free androgen index, which gives an estimate of free testosterone levels), LH, and FSH are useful in ruling out other causes of hypogonadism and monitoring treatment [15]. In older males, assessment of hypogonadism may also include semen analyses.

### 7.2. Co-Morbidities and Complications

Clinicians should be cognizantof other complications associated with obesity that may exacerbate hypogonadism, including dyslipidemia, hypertension, diabetes, and other metabolic disorders.^15^ Evaluations for bone density, cardiovascular health, sleep apnea, and mood disorders should be undertaken on a case-by-case basis. These are elaborated below.

### 7.3. Consequences of Hypogonadism

Hypogonadism is associated with many effects even beyond the reproductive system. As we have seen above, hypogonadism associated with obesity is not only associated with low testosterone, but also high estrogen, high insulin, leptin resistance among other hormonal abnormalities. Below we describe the consequences of hypogonadism that may have multiple causes and also consequences where low testosterone plays the major role. Testosterone replacement trials (TTrials) have studied the effects of restoring normal testosterone levels in 788 men with low testosterone [70]. The results of these trials are mentioned throughout the following sections as per the relationship between testosterone levels and extra-gonadal health outcomes.

#### 7.3.1. Fatigue

Fatigue can be generally defined as the type of tiredness that does not improve even after restful sleep. Being fatigued is one of the most common and debilitating symptoms of hypogonadism after mood instability and loss of muscle strength in adults. Depleted energy levels can be directly related to alterations in body composition involving muscle, fat, and lean mass, or the alerted states of hormones leading to decreased energy balance [71,72]. The particular relationship between low testosterone and fatigue exhibits a positive trend, in which energy levels decrease as testosterone levels decrease [73]. The Vitality Trial, which assessed energy levels in the men receiving testosterone therapy vs. placebo using the Functional Assessment of Chronic Illness Therapy (FACIT)-Fatigue scale, did not show significant increases in vitality in the testosterone therapy group [70]. Nevertheless, results using a vitality subscale did show significant improvements in vitality [70]. The effect of low testosterone and hypogonadism in general on the symptoms of fatigue in adolescents needs further evaluation.

#### 7.3.2. Depression

A link between depression and hypogonadism has been suggested, although associated trends between the two have not been fully understood due to inconsistent results from studies [74,75,76,77]. Some studies in older males have found that a low free testosterone is associated with a higher prevalence of depression, and conversely males with depression were up to 2.71 times more likely to have low free and total testosterone [76,77]. Results of the TTrials found a significant improvement in mood and depressive symptoms in the testosterone therapy group vs. placebo, further illustrating the association between testosterone levels and mood [70].

In adolescents, the reduction of hormones causes alterations in the physical development and body habitus during puberty and lead to emotional and psychosocial consequences. They are also at greater risk of getting bullied by their peers, leading to increased levels of depression and anxiety [59]. Symptoms of hypogonadism overlap considerably with symptoms of depression, and thus it is possible that the two are associated for this reason [77]. As mentioned, these patients experience fatigue and decreased sexual drive due to hypogonadism, but these symptoms can also be linked to depression. In some cases, it may be that these symptoms associated with hormone deficiency may be causing the individual’s mood to suffer, which eventually develops into depression. Alternatively, it may be that testosterone levels directly impact serotonin levels in the brain, thereby affecting mood regulation [78]. The sexual dimorphism observed in the prevalence of depression, with women having greater risk, suggests a role of sex hormones in the development of depression. Both low and high testosterone levels are associated with depression in women [79]. There are no data evaluating the effects of testosterone replacement on depression in adolescent males.

#### 7.3.3. Changes Associated with Low Testosterone

##### Bone Density

Adolescence is characterized by bone mass accrual, with almost 60% of the total bone mass being accumulated during puberty. Bone growth and maintenance are significantly influenced by testosterone. Androgens and estrogen (even in males) are crucial in building the skeletal system of young men and aid in preventing bone loss as an individual gets older [80]. Hypogonadism coexists with increased visceral/truncal fat, which has been shown to be associated with decreased areal bone mineral density in adults and young children [71]. More specifically, hypogonadism (from reasons other than obesity) is primarily associated with reduced bone mineral density at the spine, more so than in other areas that are similarly vulnerable to high-impact loading activities [71,81]. There is evolving data to suggest suboptimal adaptation in bone microarchitecture with increasing weight in adolescents with obesity [82], and whether low testosterone exacerbates this effect has yet to be further elucidated. Results of the Bone Trial of the TTrials showed significant increases in overall bone mineral density (BMD), BMD of spine trabecular bone, strength of the spine trabecular bone, and strength of the hip after 1 year of testosterone treatment in older men [70].

##### Decreased Libido

In postpubescent males (after pubertal completion), low testosterone levels are associated with decreased sexual desire, variable degrees of sexual dysfunction, and in some cases, erectile dysfunction [83,84,85]. Testosterone is also essential for maturation of the testis, as well as adequate production of sperm [85]. Results of the Sexual Function Trial of the TTrials showed substantial increase in sexual activity, libido, and to a lesser extent, erectile function after testosterone replacement in older men [70].

##### Cognition

Studies have shown that a mid-range of serum testosterone level corresponds with optimal cognitive performance [86,87,88]. An increase in free testosterone is associated with improved scores on visual and verbal memory, visuospatial function, and visuomotor scanning, as well as slower rates of decline in visual memory compared to men with hypogonadism [86]. One study was conducted on male Syrian hamsters to test whether the presence of gonadal hormones impacts adolescent brain development [87]. Researchers found that gonadal hormones play a role in masculinizing behavioral responses, where hamsters who were not exposed to these hormones in adolescence experienced long-lasting impairment of testosterone-induced reproductive behavior [87]. Very few studies have looked into how the presence of gonadal hormones during adolescent brain development influences adult behavior [87]. One study compared spatial cognition in males with hypogonadotropic hypogonadism acquired in adulthood with males diagnosed with idiopathic hypogonadotropic hypogonadism (IHH) before puberty [88]. Researchers found that IHH males not exposed to pubertal hormone treatment during or before adolescence were more likely to be impaired in spatial ability [88]. Hier and Crowley concluded that testosterone-mediated androgenization, specifically during pubertal development in adolescence, is essential to the development of spatial ability [88]. Results of the cognitive function trial of the TTrials found no improvement in cognitive function when measuring for delayed and immediate paragraph recall, executive function, visual memory, spatial ability, subjective memory complaints, and global cognitive function [70]. Nonetheless, the exact relationship between testosterone levels and cognitive function is not fully understood in adolescents with obesity [86].

### 7.4. Type 2 Diabetes and Cardiovascular Disease

Low testosterone levels in men are associated with insulin resistance. Hypogonadism results in an increased accumulation of visceral fat around the abdominal area, which increases the risk of developing metabolic syndrome, along with T2DM and cardiovascular disease. Men with impaired fasting glucose or glucose intolerance have biochemical evidence of hypogonadism, and prediabetic subjects are twice as likely to have low total testosterone levels compared to euglycemic controls, regardless of age [89]. Yassin and colleagues conducted an 8-year study that administered testosterone therapy to prediabetic males who had symptoms of hypogonadism and compared them to an untreated control group of males with hypogonadism. Results showed that long-term testosterone treatment had prevented progression of prediabetes to T2D in these male subjects, and most of them achieved resolution of prediabetes [89].

From a cardiovascular standpoint, in adults, testosterone deficiency is also associated with worse lipid profiles and decreased exercise tolerance [90]. Testosterone therapy in males with hypogonadism, shows favorable outcomes in lipid profile [91]. Furthermore, Adorni et al. showed that in hypogonadal men cholesterol efflux capacity (CEC) of HDL was reduced, while HDL-C remained in the normal range, demonstrating that HDL functionality is decreased and should be monitored in males with hypogonadism rather than the concentration [92]. Both glycemic control and lipid profiles should be taken into account when treating hypogonadism in males with obesity. There are no studies evaluating the associations between hypogonadism and CV outcomes in adolescents with obesity.

### 7.5. Alteration of Body Composition

Low testosterone is associated with reduced lean body mass, reduced muscle mass, and increased visceral fat. This may further contribute to an increased risk of insulin resistance and diabetes [90,93]. Testosterone has been known to stimulate growth of muscles and increase muscle strength, allowing for more muscle development during puberty in males [70]. Results from the Physical Function Trial of the TTrials found an increase in the distance walked within a given timeframe across all TTrial participants [70].

### 7.6. Quality of Life

Psychological well-being is an essential factor to consider in adolescents who suffer from hypogonadism. That is, disruptions in puberty introduce negative effects on the psychosocial development of children during the stressful time of adolescence [59]. Adolescents with hypogonadism are more likely to experience bullying and victimization, which is associated with increased levels of anxiety and depression [59]. There are no studies that evaluate the effects of optimizing serum testosterone levels on quality of life in adolescents with obesity.

### 7.7. Long-Term Fertility

Male obesity has detrimental effects on sperm that are independent of the HPG axis suppression discussed earlier [94]. Excess adipose tissue around the scrotal area increases the local temperature there [94]. Sertoli cells that support spermatogenesis are temperature-sensitive cells whose activity is dependent on a temperature that is less than body temperature (<37 °C) [94]. The increased temperature resulting from excess adipose hinders sertoli function and thus impairs sperm production, contributing to subfertility [94]. There are also other mechanisms of reduced fertility in males that are associated with obesity, including inflammation that has been shown to reduce sperm quality and motility, reactive oxygen species that may cause, D.N.A.; protein, and membrane damage in sperm cells, as well as epigenetic changes that contribute to subfertility [16,94,95,96,97,98,99,100]. These effects potentially contribute to long term subfertility for adolescent males with obesity who continue to have excess adipose tissue and experience hypogonadism in adulthood.

### 7.8. Impact on the Next Generation

A limited number of studies have looked into the effects of paternal obesity on the metabolic health of offspring, and it has been suggested that offspring from fathers with obesity are more likely to suffer from obesity and other metabolic conditions as well [101,102,103,104,105]. Furthermore, Fullston et al. conducted a study that administered a high-fat diet to induce obesity in male mice to observe the effects of paternal obesity on offspring health outcomes after mating with normal-weight females [103]. Results report negative effects on the reproductive health of offspring following 2 generations, which can potentially have transgenerational implications in humans as well [103].

## 8. Treatment of Hypogonadism

### 8.1. Weight Loss

Weight loss is associated with increased free and total testosterone levels in males with hypogonadism [106,107,108,109]. It is also associated with increased SHGB and decreased estrogen levels [106,107,108]. These benefits occur regardless of the method of weight loss, whether through lifestyle/dieting or bariatric surgery, but they are more pronounced following the latter, since more weight loss is often achieved through bariatric surgery than lifestyle changes [107,109,110]. Multiple studies show that the increase in, F.T.; TT, and SHBG are directly related to the degree of weight loss [108]. A recent meta-analysis found that in individuals with obesity who subsequently lost weight, TT rose more significantly in younger males than in older males, further emphasizing the importance of weight loss interventions to treat hypogonadism in adolescent males with obesity [106]. Weight loss in men with hypogonadism has also been shown to improve erectile function [109,110]. Weight loss medications also contribute to the reversal of hypogonadism, and their effects are most pronounced when used as adjunctive therapy to bariatric surgery [110,111,112]. Even short-term lifestyle modifications in combination with metformin have been shown to result in increased TT and FT levels [111]. Another study found that adding liraglutide, a glucagon-like peptide 1 (GLP-1) agonist commonly used for weight loss, to a therapeutic regimen of lifestyle changes, metformin, and testosterone increases testosterone levels and enhances sexual function more than treatment with testosterone alone [112]. The benefits of weight loss medications on obesity-associated hypogonadism in adolescent males still requires investigation.

### 8.2. Testosterone

Testosterone treatment is widely used for adult men with obesity. It has been shown to have the beneficial effects of reducing visceral fat, treating metabolic syndrome, and improving insulin sensitivity [89,93]. Hormonal replacement in men with hypogonadism increases sexual interest, sexual function, and the frequency of spontaneous erection [113,114,115,116]. In addition, treatment has also been seen to improve areal and volumetric bone mineral density and bone strength in the hip and spine in older men with hypogonadism [116]. Furthermore, it is important to note that the literature primarily captures testosterone treatment of hypogonadism among adult subjects, and there is limited information on how these treatments impact adolescent males.

A study was done to assess the effects of low-dose testosterone treatment in adolescents diagnosed with delayed puberty within a four-month timeframe. Researchers concluded that fat free mass increases as fat mass and leptin levels decreased [117]. In addition, it was found that insulin clearance increases, whereas insulin sensitivity of glucose metabolism did not change in these adolescents [117]. Low testosterone levels are also known to impair bone mineralization during puberty [118]. One study showed that this effect was reversed with testosterone treatment in adolescent patients with hypogonadotropic hypogonadism, allowing for an improved bone mineral status even after puberty [118].

An important side effect of exogenous testosterone as therapy is decreased testicular production of testosterone [119,120]. Exogenous testosterone provides a negative feedback on, L.H.; which suppresses its release and thus suppresses testosterone production [121]. Also, exogenous testosterone administration in adolescent males may result in premature closure of the growth plates of bones [122]. Estrogen is a key regulator of growth plate fusion, and the additional estrogen that results from aromatization of the exogenous testosterone may play a role in this effect [122]. Because adolescents’ growth plates are not yet fused, testosterone first causes a brief period of growth in long bones, and then premature closure, which may result in overall decreased height [122]. Other possible untoward side effects of testosterone therapy include increased, L.D.L.; decreased, H.D.L.; renal failure, and liver damage [84,120].

The administration of androgens in adolescent males remains controversial, and the pubertal progress and growth potential of the individual must be considered in order to allow for normal linear growth and epiphyseal maturation [123,124].

### 8.3. Clomiphene Citrate

As mentioned, a concern of exogenous testosterone therapy is its suppression of endogenous testicular production of testosterone, leading to suppression of spermatogenesis and thus contributing to infertility, which is of particular relevance to adult males [119,125] Clomiphene citrate is a selective estrogen receptor modulator (SERM) that competes with estrogen to bind to estrogen receptors on the hypothalamus and pituitary gland [125]. This reduces estrogen’s negative feedback effects on the HPG axis, leading to increased production of GnRH in the hypothalamus and LH and FSH in the pituitary gland [125]. The increase in gonadotropic hormones then result in increased testosterone production in the testes [125]. A recent randomized, double-blind, placebo-controlled study showed clomiphene citrate’s efficacy in treating obesity-related hypogonadism in 78 adult men [126]. The study found significant improvements in hormone levels (increased, T.T.; FT, LH, FSH, and SHGB), body composition (increased muscle mass, lean mass, and fat-free mass), and sexual function (improvement of erections) [126]. Many studies show similar findings of improved testosterone levels in males with hypogonadism undergoing clomiphene citrate therapy, and its efficacy has been illustrated in adult men from across a wide age range (18–70 years) [120,121,125,127,128,129,130,131].

As more patients are using clomiphene citrate, for male hypogonadism as well as for treatment of other reproductive diseases, it has become increasingly important to understand its long-term effects. The possible side effects of clomiphene citrate include enlarged male breasts, hypertension, abdominal discomfort, cataracts, and weight gain [120,127]. One literature review gathered data from 32 articles and found that prolonged use of clomiphene citrate has some level of genotoxicity (ability to cause mutations in genetic material of cells), cytotoxicity, and, when used in women, increased risk of certain cancers [130]. The genotoxic and cytotoxic effects were reproduced in bacterial, animal, and human cells [130], but more studies are needed to determine the mechanism of toxicity [130].

Another benefit of clomiphene citrate therapy is a reduced risk of developing erythrocytosis/polycythemia (increased production of red blood cells), one of the most common side effects of testosterone therapy [132,133]. A study comparing 188 men on clomiphene citrate and 175 men on testosterone illustrates this, in which both treatments showed similar efficacy in restoring testosterone levels, but clomiphene citrate treatment resulted in a significantly lower rate of polycythemia compared to testosterone treatment (1.7% vs. 11.2%) [132]. Furthermore, clomiphene citrate therapy is convenient for many, as it can be administered orally [125]. The use of clomiphene citrate to increase endogenous testosterone production in adolescent males with obesity still needs evaluation.

### 8.4. Human Chorionic Gonadotropin (hCG)

HCG is a hormone naturally produced by the placenta during pregnancy that has been used as treatment in male hypogonadism to induce testosterone production and spermatogenesis [125,134]. This is possible due to the similarity in structure of hCG to, L.H.; in which hCG can stimulate Leydig cells to produce testosterone [125]. Like clomiphene citrate, one benefit of hCG over exogenous testosterone is that it preserves the endogenous production of testosterone and maintains an adequate intratesticular testosterone level, which is crucial for the health of Sertoli cells and thus fertility [125]. Studies have shown increased sperm counts and testosterone levels in men with hypogonadism who receive hCG therapy [134]. Nonetheless, although hCG treatment induces spermatogenesis in patients with hypogonadism, studies show that over time, sperm count and thus spermatogenesis decreases with hCG therapy alone [134]. This is most likely due to the lack of improvements in, F.S.H.; which is needed to sustain long-term spermatogenesis [134]. Some drawbacks of hCG treatment are its high cost and its administration through injection [125]. The possible side effects of hCG are similar to those of clomiphene citrate, but they also include headaches, tiredness, and feet swelling [120]. As before, much of the data for the efficacy and side effects of these treatments comes from adult males with infertility. Their use in adolescents with obesity has not yet been investigated.

## 9. Conclusions

Hypogonadism in male adolescents with obesity is increasing as obesity levels continue to rise worldwide. Obesity plays a major role in hypogonadism through complex interactions between gonadal hormones, excess adipose tissue aromatase activity and adipocyte hormone production, and inflammatory markers, all of which ultimately results in decreased testosterone production. The effects of low testosterone in male adolescents can be substantial, and they include abnormal puberty and development of sexual characteristics, advanced bone age, shorter adult height, subfertility, decreased bone and muscle mass, cardiovascular risk, fatigue, and depression, among others. Male adolescents may present with few symptoms of hypogonadism, and thus new markers and methods for diagnosis of male hypogonadism are continuously being updated. Recognition and monitoring of hypogonadism in adolescent males with obesity is the first step in developing effective strategies to tackle this disease. The literature on hypogonadism in adolescent males with obesity is limited. This scarcity in data is most notable for the efficacy of different forms of treatment. Thus, further studies on the pathophysiology, outcomes, evaluation, and treatment of hypogonadism in male adolescents with obesity are needed.

## Figures and Tables

**Table 1:** Syndromes associated with hypogonadism [60,61,62,63,64,65,66,67,68,69].

Syndrome	Description and Main Symptoms	Associated Genes	Other Co-occurring Symptoms
Bardet-Biedl syndrome	The mutation that occurs in one of the many genes associated with this condition introduces issues with cellular communication, due to the malfunction of cilia present in cells. Symptoms involved in this disorder vary, but some of the most common are visual impairment by retinal abnormalities, obesity, and kidney malfunctions.	Mutations in at least one of 19 different genes (BBS genes): ARL6, BBIP1, BBS1, BBS2, BBS4, BBS5, BBS7, BBS10, BBS12, CEP290, IFT27, LZTFL1, MKKS, MKS1, SDCCAG8, TRIM32, TTC8, WDPCP	Developmental delay Intellectual disability and cognitive delay Polydactyly (presence of extra fingers or toes) Dental abnormalities Loss of the sense of smell
X-linked adrenal hypoplasia congenita	While this disorder primarily impacts males, it primarily targets adrenal glands and other endocrine tissues in the body that produce hormones to regulate functions in the body. The primary sign of this condition is adrenal insufficiency, in which the adrenal glands do not produce enough hormones.	NR0B1	Adrenal failure Shortage of male sex hormones Delayed puberty and potential infertility
Xp22.3 contiguous gene deletion syndrome	This gene syndrome is caused by an interstitial deletion in Xp22.3. Associated symptoms include intellectual disabilities, short stature, and dysmorphic features.	Xp22.3 microdeletion	Skeletal abnormalities Ichthyosis ADHD Chondrodysplasia punctate (disorder of cartilage and bone development)
Prader-Willi Syndrome	A genetic disorder resulting from an abnormality at chromosome 15 around the time of conception. This condition impacts metabolism, growth, and cognitive function. Symptoms can change as an individual ages, but the most common involve dysfunction of the hypothalamus, growth hormone deficiency, and obesity,	Loss of paternal 15q11.2	Developmental delay Low muscle tone Cryptorchidism/microphallus in males Intellectual disability
CHARGE syndrome	This condition is caused by mutations in the CHD7 gene which functions to make a protein involved in gene expression. This introduced glitch in gene expression causes the common symptoms that include coloboma, heart defect, atresia choanae, restricted growth, genital abnormality, and ear abnormality.	CHD7	Cleft lip or palate Kidney abnormalities Genital abnormalities Scoliosis
Gordon-Holmes Syndrome	This condition involves mutations primarily in the PNPLA6 and RNF216, which are involved in neural processes observed in synaptic connections and the release of hormones. Common symptoms involve developmental delay in puberty and neurological problems.	OTUD4 PNPLA6 RNF216 STUB1	Dementia Cerebellar ataxia (balance and coordination difficulties) Speech Difficulties
Combined Pituitary Hormone Deficiency	A sporadic condition that reduces the amount of different hormones produced by the pituitary gland, which may affect the development of different parts of the body. One of the primary symptoms associated with this syndrome is hypothyroidism.	HESX1 LHX3 LHX4 POU1F1 PROP1	Weight gain and fatigue (associated with hypothyroidism) Infertility Delayed or absent puberty Cortisol deficiency (causing a weakened immune system)
HFE-associated hereditary hemochromatosis	Characterized by the abnormally high absorption and storage of iron in the liver, pancreas, heart, joints, and anterior pituitary gland. This overload of iron can eventually damage tissues and organs. Some common symptoms are abdominal pain, diabetes mellitus, lethargy, and arthralgias,	HFE	Arthritis Liver disease hypogonadism Congestive heart failure Skin discoloration
Congenital Hypogonadotropic Hypogonadism	A rare condition caused by a deficiency or insensitivity to gonadotropin-releasing hormone (GnRH). This monogenic disorder is characterized through hypogonadotropic hypogonadism, in which an individual experiences incomplete or absent puberty and infertility.	GNRH, GNRH1, KISS1R, KISS1, TACR3, IL17RD, FGFR1, PROKR2	Cleft lip or palate Dental agenesis Ear abnormalities Congenital hearing impairment Skeletal abnormalities Renal agenesis Bimanual synkinesia
Kallmann Syndrome	This condition is a form of hypogonadotropic hypogonadism characterized by incomplete or absent puberty and an impaired sense of smell. At puberty, most individuals do not develop secondary sexual characteristics, and potentially become infertile.	KAL1, FGFR1, FGF8, CHD7, HS6ST1, SOX10, SEMA3A, WDR11, IL17RD, PROKR2, PROK2, FEZF1	Olfactory bulb hypoplasia Aplasia on brain Renal agenesis Micropenis and/or undescended testes Dental agenesis Skeletal abnormalities Cleft lip or palate Hearing impairment Bimanual synkinesia
